# Supplementary figures and images for: Exploring Epigallocatechin-3-Gallate Autoxidation Products: Specific Incubation Times Required for Emergence of Anti-Amyloid Properties
Source: Antioxidants (Basel). 2022 Sep 23;11(10):1887. doi: 10.3390/antiox11101887 (PMC9598636; doi:10.3390/antiox11101887)

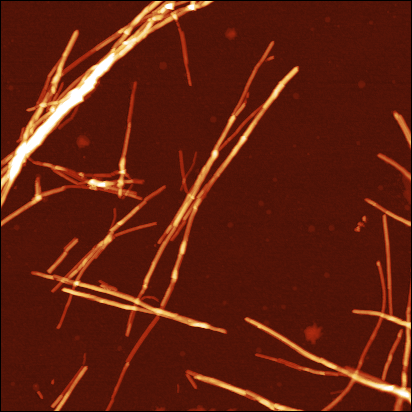

Supplement: Supplementary file 1 [file antioxidants-11-01887-s001.zip › AFM image of sample 1.tiff]

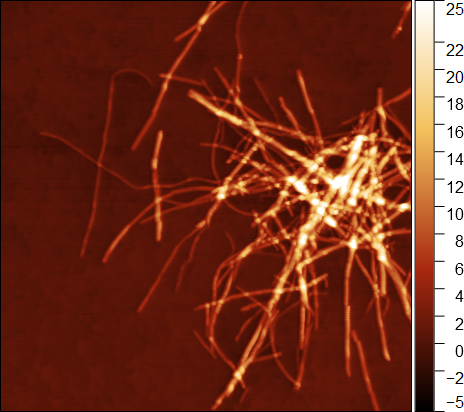

Supplement: Supplementary file 1 [file antioxidants-11-01887-s001.zip › AFM image of sample 24.tiff]
